# Case report: Three-dimensionally printed patient-specific acetabular cage for revision surgery of aseptic loosening in a dog with micro total hip replacement

**DOI:** 10.3389/fvets.2022.915639

**Published:** 2022-10-19

**Authors:** AhRan Kang, Haebeom Lee, Yoonho Roh, Daehyun Kim, Seong Mok Jeong, Jaemin Jeong

**Affiliations:** ^1^College of Veterinary Medicine, Chungnam National University, Daejeon, South Korea; ^2^Division of Small Animal Surgery, Department of Clinical Veterinary Medicine, Vetsuisse Faculty, University of Bern, Bern, Switzerland

**Keywords:** canine, micro total hip replacement, aseptic loosening, revision surgery, patient-specific acetabular cage

## Abstract

A 2-year-old castrated male Pomeranian dog was presented for regular follow-up after micro total hip replacement (mTHR) 16 months prior to presentation. Clinically, the dog did not show any noticeable lameness of the left hindlimb, except for external rotation during walking. However, radiographic findings, namely rotation and medialization of the acetabular cup with a periprosthetic lucent line and bone formation medial to the acetabulum, were interpreted as aseptic loosening of the acetabular component. Because the dog was incompatible with the conventional THR revision method owing to severe bone defects in the acetabulum, a patient-specific titanium acetabular cage prosthesis with biflanges and four cranial and one caudal screw hole was designed for revision surgery. A custom-made acetabular cage was prepared, and it had a 12-mm polyethylene cup fixed with polymethylmethacrylate bone cement and positioned in the acetabulum. After the custom-made acetabular cage was anchored to the pelvic bone with the five cortical screws, reduction of the prostheses was achieved smoothly. The dog showed almost normal limb function without external rotation of the left hindlimb 2 weeks postoperatively. Bone remodeling and stable implant position were noted on radiographic images 3 years after revision surgery, with no evidence of loosening. Based on the clinical outcomes, the use of a custom-made acetabular prosthesis can be an effective treatment option for revision arthroplasty in acetabula with severe bone loss and structural changes in small-breed dogs.

## Introduction

Micro total hip replacement (mTHR) is a salvage surgical procedure that replaces the affected coxofemoral joint in which degenerative joint diseases, luxation, femoral head and neck fracture, or Legg-Calve-Perthes disease is present in small-breed dogs. The prostheses for mTHR surgery were developed in 2005, relatively recently compared to standard THR implants for large-breed dogs. In small dogs and cats weighing < 12 kg, mTHR can be performed to improve quality of life by retaining biomechanical function and eliminating pain in the affected coxofemoral joint (1).

Few studies have reported the outcomes and complications of mTHR in small dogs and cats with coxofemoral disease (2–5). Although the outcome has been successful in more than 90% of cases, the reported complications of mTHR include coxofemoral luxation, infection, cortical wall penetration, femoral fracture, aseptic loosening, sciatic neuropraxia, medial patellar luxation that developed after THR, and femoral medullary canal infarction. However, only a few reports on the treatment of complications have been published (2, 3). Aseptic loosening of femoral and acetabular components is the most common late complication following cemented THR in dogs (4, 6–9) with an overall incidence of 3.0 and 5.5%, respectively. It accounts for more than half of the implants in post-mortem investigations (10, 11).

Aseptic loosening is mainly due to wear debris-mediated osteolysis. It is a major cause of acetabular implant failure in THR that requires revision surgery along with removal of the periprosthetic fibrous membrane (9). The main revision strategy for acetabular cups reported in dogs and humans is implant replacement (6, 12, 13) if early detection can be achieved and only when little structural change and adequate bone stock exist. However, in thin and small-boned animals, such as small-breed dogs and cats, or patients with significantly advanced osteolysis and inadequate bone support, this conventional method is challenging.

Alternatively, structural augmentation may also be necessary. For acetabular reinforcement, methods using plate and screw fixation of polymethylmethacrylate (PMMA) (14) and autogenous bone blocks from the excised femoral head, iliac wing, or bone allograft have been reported (15). Often, the explantation of loose implants may be necessary as a last treatment option. Recently, as a way of overcoming these structural limitations, three-dimensional (3D)-printed patient-specific implants for the revision of THR with insufficient acetabular bone stock have been reported in humans (16–18) and large-breed dogs (19). However, revision strategies for mTHR in small-breed dogs have not yet been reported clinically.

The purpose of this case report was to describe a revision strategy and prognosis for aseptic cup loosening in mTHR using a 3D-printed patient-specific acetabular cage.

## Case description

**Figure 1:**
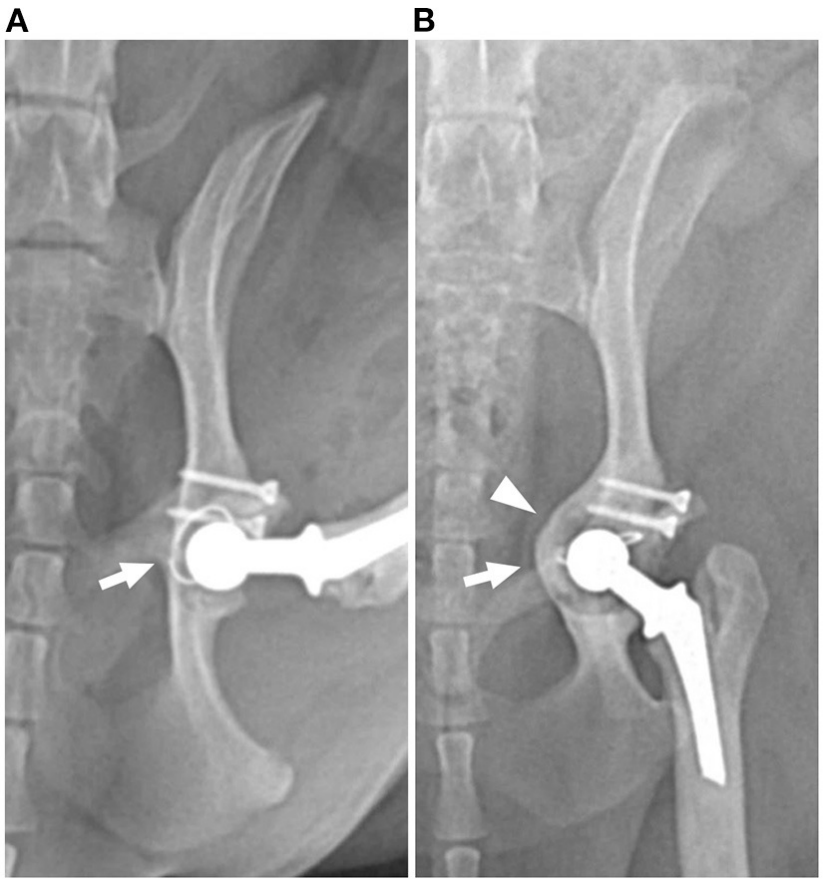
Postoperative ventrodorsal radiographs of the left hip showing evidence of loosening with rotation (arrow) and medialization (arrowhead) of the acetabulum and cemented cup implant. **(A)** Immediately and **(B)** 16-month postoperative radiographs.

A 2-year-old, 10.1-kg, castrated, male, Pomeranian dog was presented for regular follow-up after mTHR. The patient had a history of bilateral hip dysplasia and had undergone left mTHR at our institution 16 months prior to presentation, using a previously reported procedure (2). A micro total hip replacement (Biomedtrix, Boonton, NJ, USA) system with a #2 stem and 12-mm cup had been implanted, and cranial pole augmentation of the acetabulum was performed using PMMA bone cement with screw fixation (Figure 1A).

At presentation, the dog did not show any noticeable lameness of the left hindlimb, except for external rotation during walking. The measured thigh circumferences of the left and right hindlimb were 21.5 and 20.3 cm, respectively. There was no evidence of infection in this patient.

Radiographs (Figure 1B) revealed severe bone loss and medialization of the medial acetabular wall. The position of the cup implant was changed compared to previous radiographs and cement-implant debonding was suspected. Radiographic findings and clinical evidence were interpreted as aseptic loosening of the acetabular component.

**Figure 2:**
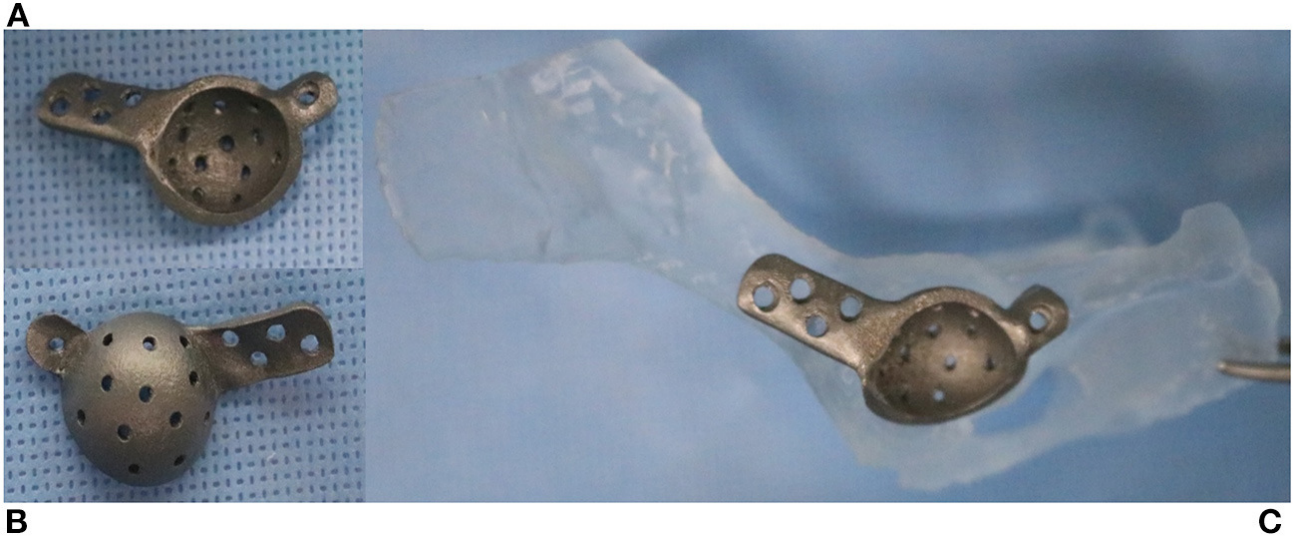
Three-dimensionally printed pelvic bone and acetabular implant. Lateral **(A)** and medial **(B)** aspects of the cage. Two flanges provide screw holes to achieve initial fixation to the ilium and ischium **(C)**. The medial side of the implant has a porous surface for long-term biological fixation.

The patient was incompatible with the conventional THR revision method because of its small size, anatomical changes, and insufficient acetabular bone stock. Preoperative computed tomographic images of the pelvis were obtained by using a 16-detector row scanner (Alexion^TM^, Canon Medical Systems, Japan) with the following parameters: 150 mA, 120 kVp, 0.75 s rotation time, 1.0 mm slice thickness, and collimation beam pitch of 0.938. All digital imaging data were analyzed using the Mimics 19.0 software (Materialise, Leuven, Belgium) to assess the anatomy of the pelvic bone and residual acetabular bone stock. The STL file of pelvic bone was imported into 3-Matics 11.0 software (Materialise, Leuven, Belgium) and a patient-specific titanium acetabular prosthesis was designed. The acetabular prosthesis produced by SLM 280HL (SLM Solutions GMbH, Germany) had bi-flanges with four cranial and one caudal screw hole, for fixation to the ilium and ischium (Figure 2).

Preoperative rehearsal was performed using the 3D-printed pelvic model and a patient-specific implant to confirm the position of the cage and the angle of the acetabular cup for fixation during the actual procedure (Figure 2C).

The dog was premedicated with hydromorphone (0.05 mg/kg, IV) and midazolam (0.2 mg/kg, IV). General anesthesia was induced with propofol (6 mg/kg, IV) and maintained with isoflurane. Analgesia was provided by constant rate infusion of remifentanil (0.1–0.3 μg/kg/min). Cefazolin (22 mg/kg, IV) was administered 30 min before incision and repeated every 90 min. A craniolateral approach to the left hip joint was performed.

A custom-made acetabular cage was prepared, and it had a 12-mm polyethylene cup cemented with PMMA at a 45° angle of lateral opening and 14° retroversion angle of cup prosthesis, as planned in rehearsal surgery.

Loosening and inner surface wear of the previously inserted acetabular polyethylene cup were identified during surgery. Osteolysis of the periprosthetic area was confirmed, and fibrotic tissue was debrided by curettage. The primary implanted cup, cement mantle, and screws used for cranial pole augmentation were removed. An autogenous cancellous bone graft harvested from the ipsilateral proximal humerus was applied to the medial area of the acetabulum, where bone loss was evident. The patient-specific implant was positioned in the acetabulum and anchored to the pelvic bone with five 1.5-mm cortical titanium screws. After reduction with the previously inserted femoral head component (8 mm +2), the surgical site was lavaged with sterile saline, and a surgical site swab was taken for microbial culture. The site was routinely closed.

**Figure 3:**
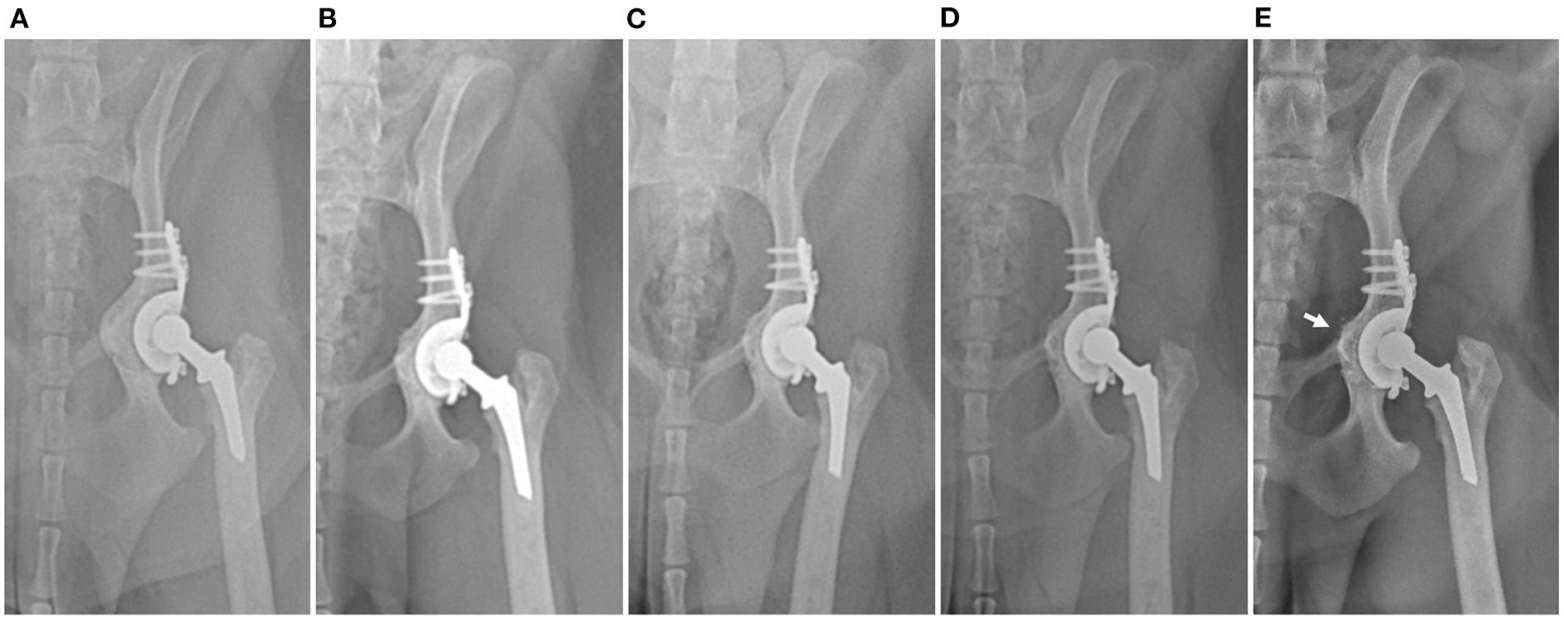
Postoperative serial craniocaudal radiographs of the left hip with cage at subsequent follow-ups at **(A)** immediately postoperatively and **(B)** 5 months, **(C)** 15 months **(D)** 20 months, and **(E)** 39 months postoperatively. No evidence of loosening or change in implant position and gradual remodeling of medialized pelvic bone (arrow) can be observed on the radiographs.

Radiographs were obtained immediately postoperatively and revealed the intended position of the acetabular cage. The angle of lateral opening, retroversion, and inclination angle of a revised 12-mm polyethylene cup were 51°, 21°, and 27°, respectively. Cefazolin was administered as a postoperative antibiotic for 2 weeks without bacterial culture growth. Surgical wound healing was uneventful. Limb function progressively improved without complications. Two weeks postoperatively, the dog showed almost normal limb function without external rotation and was discharged. Bone remodeling and stable position of both acetabular cage and polyethylene cup were noted on radiographic images with no evidence of loosening at 2, 5, 15, and 2 years after revision surgery (Figure 3). At the 33-month postoperative visit, craniodorsal luxation of mTHR caused by a traumatic event was diagnosed. Closed reduction was performed successfully, and the dog had normal limb function during the study period without implant loosening 39 months postoperatively.

## Discussion

This study describes acetabular revision surgery using a 3D-printed patient-specific titanium acetabular cage in a dog with severe bone loss and deformity of the acetabulum following mTHR surgery caused by aseptic cup loosening. To our knowledge, this is the first report on the application of a customized acetabular implant for mTHR revision. Although revision surgery was required to address aseptic loosening in our case, the patient did not show unsatisfactory clinical outcomes, including significant muscle atrophy and pain on passive movement, frequently seen after femoral head and neck ostectomy (20). Further, the clinical outcome after revision surgery showed excellent hind limb function until 3 years of follow-up.

In this dog, cup loosening at 16 months' follow-up was considered to be related to mechanical loss due to the failure of cement fixation between the bone and acetabular cup. The bonding of the cement mantle to the bone or implant surface may influence micromotion and mechanical instability of the acetabular cup. Maldistribution of the load to the cup surface due to mechanical loss may lead to the acceleration of wear of the polyethylene cup (21). As the cement mantle layer transmits a load between the prosthesis and bone, it is important to ensure during implant insertion that the thickness of the cement mantle can withstand long-term force (22). A recommended PMMA mantle thickness of >2 mm is usually considered ideal for humans and large-breed dogs (23, 24). However, the criteria for mantle thickness in mTHR for small dogs have not been studied, and Liska (2) have obtained a mantle thickness of < 2 mm. The patient in this case was also a small dog, and a 2-mm cement mantle thickness could not be achieved at the time of surgery. The inevitable cement mantle thickness of < 2 mm in small dogs may provide insufficient mechanical fixation, but further research on biomechanical testing or extensive long-term follow-up is necessary.

Furthermore, the patient in our study was already overweight at the time of the initial mTHR and had gained weight gradually after surgery. A relatively small prosthesis was implanted in the dog because of its small bone size compared with its body weight, and the patient was highly active. This would have increased the load on the implants and worsened wear, which can induce mechanical and biological loss of fixation that could have contributed to aseptic loosening in this dog.

The conventional revision method of insertion of a larger cup for the acetabular component was difficult because of the patient's small size. The anatomy of the pelvis had changed as a result of aseptic loosening, so the acetabular cup could not be placed in its normal position. In human medicine, if the bone stock in the acetabulum is insufficient, a patient-tailored cage is used for THR surgery (16–18). Recently in veterinary medicine, Castelli et al. (19) reported a single case using a similar 3D-printed patient-specific acetabular implant in large-breed dogs. This type of implant has the advantage of being able to be fixed stably to the pelvic bone using screws, which provides initial stability for structural support with a spatial bridge function in the bone defect site. In addition, it ensures that the acetabular component is placed at a biomechanically suitable site (25, 26). Despite these advantages, no custom-made implants have been reported for mTHR revision in small dogs.

In this case study, we modeled the patient's pelvic bone and produced a patient-specific implant for the revision of the acetabular component. It was designed to fit exactly into the patient's acetabulum, and the surface had a porous texture that was three-dimensionally designed and printed to induce biological integration with the bone, thereby providing long-term stability of the implant. Three years postoperatively, osteointegration of the cage surface with the acetabular bone was observed. Using this implant, the prosthesis could be effectively implanted in a small dog with large bone loss and structural changes of the acetabulum. Medialized bone proliferation was remodeled during a 3-year follow-up, showing no osteolytic change, and the device was in a stable position.

The limitations of our study include the fact that it was based on a single case and that the biomechanical aspect of the implant was not evaluated, even though it showed excellent outcomes. Additionally, we suspected aseptic loosening through laboratory and culture tests. However, previous studies reported that the sensitivities of blood exams and tissue culture tests for prosthetic joint infection were much lower than other modifications (27, 28). It would have been necessary to rule out the microorganisms through advanced tests, including polymeric chain reaction-based methods or radio-labeled white blood cell scintigraphy (28–30). Although we did not completely rule out septic loosening, we successfully addressed the loosening implant through a one-stage revision using a 3D-printed patient-specific acetabular cage. Lastly, a follow-up period of 3 years may not be sufficient for evaluating extensive long-term results.

## Conclusion

This case report describes a revision strategy for mTHR using a 3D-printed patient-specific acetabular cage in a dog with abnormal bone remodeling of the acetabulum, severe bone loss, and structural changes caused by aseptic loosening. Based on the clinical outcomes, the use of a custom-made acetabular prosthesis can be an effective treatment for aseptic acetabular component loosening. Further studies on the biomechanical features of implants are required.

## Data availability statement

The raw data supporting the conclusions of this article will be made available by the authors, without undue reservation.

## Ethics statement

Ethical review and approval was not required for the animal study, because this study is a case report of examinations and surgery performed for the purpose of treatment of patients, and no action contrary to treatment was performed. Written informed consent was obtained from the owners for the participation of their animals in this study.

## Author contributions

AK and JJ performed clinical management of the case, wrote, and edited the manuscript. HL performed all of the surgeries. JJ and HL contributed to the conception of the case report and revised the manuscript. YR, DK, and SJ supervised the clinical management of the case. All authors contributed to preparation and final approval of the manuscript.

## Conflict of interest

The authors declare that the research was conducted in the absence of any commercial or financial relationships that could be construed as a potential conflict of interest.

## Publisher's note

All claims expressed in this article are solely those of the authors and do not necessarily represent those of their affiliated organizations, or those of the publisher, the editors and the reviewers. Any product that may be evaluated in this article, or claim that may be made by its manufacturer, is not guaranteed or endorsed by the publisher.
